# *CFTR* founder mutation causes protein trafficking defects in Chinese patients with cystic fibrosis

**DOI:** 10.1002/mgg3.258

**Published:** 2016-11-13

**Authors:** Gordon K. C. Leung, Dingge Ying, Christopher C. Y. Mak, Xin‐Ying Chen, Weiyi Xu, Kit‐San Yeung, Wai‐Lap Wong, Yoyo W. Y. Chu, Gary T. K. Mok, Christy S. K. Chau, Jenna McLuskey, Winnie P. T. Ong, Huey‐Yin Leong, Kelvin Y. K. Chan, Wanling Yang, Jeng‐Haur Chen, Albert M. Li, Pak C. Sham, Yu‐Lung Lau, Brian H. Y. Chung, So‐Lun Lee

**Affiliations:** ^1^ Department of Paediatrics and Adolescent MedicineLKS Faculty of MedicineThe University of Hong KongHong KongHong Kong; ^2^ Department of PsychiatryLKS Faculty of MedicineThe University of Hong KongHong KongHong Kong; ^3^ HKU Shenzhen Institute of Research and InnovationSchool of Biomedical SciencesThe University of Hong KongHong KongHong Kong; ^4^ Department of Paediatrics and Adolescent MedicineQueen Mary HospitalHong KongHong Kong; ^5^ NHS LothianLothian NHS BoardEdinburghUK; ^6^ Department of GeneticsKuala Lumpur HospitalKuala LumpurMalaysia; ^7^ Department of Obstetrics and GynaecologyTsan Yuk HospitalHong KongHong Kong; ^8^ Department of PaediatricsFaculty of MedicineThe Chinese University of Hong KongHong KongHong Kong; ^9^ Department of Paediatrics and Adolescent MedicineThe Duchess of Kent Children's Hospital at Sandy BayHong Kong

**Keywords:** Bronchiectasis, Chinese, cystic fibrosis, founder mutation, trafficking defects

## Abstract

**Background:**

Cystic fibrosis (CF) is a rare condition in Asians. Since 1985, only about 30 Chinese patients have been reported with molecular confirmation.

**Method:**

Using our in‐house next‐generation sequencing (NGS) pipeline for childhood bronchiectasis, we identified disease‐causing *CFTR* mutations in CF patients in Hong Kong. After identifying p.I1023R in multiple patients, haplotype analysis was performed with genome‐wide microarray to ascertain the likelihood of this being a founder mutation. We also assessed the processing and gating activity of the mutant protein by Western hybridization and patch‐clamp test.

**Results:**

Molecular diagnoses were confirmed in four patients, three of whom shared a missense mutation: *CFTR*:c.3068T>G:p.I1023R. The results suggested that p.I1023R is a founder mutation in southern Han Chinese. In addition, the processing and gating activity of the mutant protein was assessed by gel electrophoresis and a patch‐clamp test. The mutant protein exhibited trafficking defects, suggesting that the dysfunction is caused by reduced cell surface expression of the fully glycosylated proteins.

**Conclusion:**

Together with other previously reported mutations, the specific founder mutation presented herein suggests a unique *CFTR* mutation spectrum in the southern Chinese populations, and this finding has vital implications for improving molecular testing and mutation‐specific treatments for Chinese patients with CF.

## Introduction

**Figure 1:**
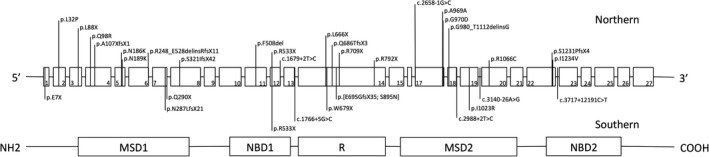
*CFTR* mutation spectrum in Chinese CF patients (findings from this study and previous studies reported in both English and Chinese medical literatures from 1985 to 2016). Only variants observed in patients with complete molecular diagnosis were included. The upper panel refers to CFTR mutations identified from Northern China (i.e., Beijing), while the lower panel refers to CFTR mutations identified from Southern China (i.e., Sichuan, Taiwan and Hong Kong). The geographical origins of mutations were determined according to either the information provided from the manuscript on patient recruitment, or the institute address of the corresponding authors. *CFTR* mutations from two reports (Zielenski et al. 1995; Wagner et al. 1999) were not included as the corresponding institute was located outside China.

Cystic fibrosis (CF) (OMIM 219700) is a common autosomal recessive genetic disorder in Caucasian populations with an incidence of approximately 1 in 2500–3500 newborns (Southern et al. 2007; Salvatore et al. 2011). Compared with Western countries, CF is a considerably rarer condition in Asia. For example, the incidence of CF is approximately 1 in 350,000 in the Japanese population (Yamashiro et al. 1997). The condition is caused by mutations in the coding gene of the membrane glycoprotein, cystic fibrosis transmembrane conductance regulator (CFTR), which is responsible for regulating the flow of chloride ions across the epithelial membrane. To date, the number of documented disease‐causing mutations in *CFTR* (RefSeq NM_000492.3) exceeds 2000 (Cystic Fibrosis Mutation Database 2016). However, molecular confirmation of mutation has only been reported for 32 Chinese patients from 1985 to 2016 (see Fig. 1 and Table S1). One explanation for the relatively high incidence of CF in the West is the founder effect of certain mutations (Castellani et al. 2008). The most common mutation, p.Phe508del (∆F508), accounts for greater than two‐thirds of CF alleles worldwide, though it is not a predominant mutation in Asian CF patients (Wong et al. 2003). Although founder mutations have not yet been identified in the Chinese population, among the *CFTR* mutations found in the Chinese population, the novel variant c.3068T>G:p.Ile1023Arg (p.I1023R) was identified in a pair of siblings with CF from Taiwan (Ho et al. 2007). Here, we describe the identification of four additional patients with the p.I1023R variant in southern China, and our results suggest that p.I1023R is a Chinese‐specific founder mutation that leads to trafficking defects and reduced expression of functionally mature CFTR protein.

## Materials and Methods

### Patient recruitment

This study was approved by the HKWC‐HKU Institutional Review Board. Informed consent was obtained from patients (or parents) after clinical consultation. Twenty‐one patients diagnosed with early‐onset bronchiectasis were referred to our unit for clinical evaluation and a sweat test. Four patients with available genomic DNA presented a strong clinical suspicion of CF and were sweat‐test positive (chloride ion concentration >60 mmol/L). Molecular diagnoses were performed for all patients.

### Experimental workflow

For a detailed description of the workflow, please refer to the supplementary data (Appendix S1).

## Results

### Detection of disease‐causing mutations in *CFTR*

Four unrelated Chinese patients from Hong Kong were positive for *CFTR* mutations, of whom three harbored a p.I1023R mutation. These patients were all compound heterozygotes with p.I1023R and one other disease‐causing mutation. Through personal communication, we identified another nonconsanguineous southern Chinese CF patient from Malaysia with a homozygous p.I1023R mutation confirmed by Sanger sequencing in our laboratory. Clinical and genetic data for the patients are summarized in Table 1.

**Table 1:** Clinical presentation of Chinese patients with molecularly confirmed CF. Organisms in **bold** indicate bacteria isolated from first‐time culture. y.o., year old; m.o., month old; Age at Dx, age at diagnosis^a^; PFT, pulmonary functional test; MI, meconium ileus; PI, pancreatic insufficiency^a^; HRCT, high‐resolution computed tomography; Ac, *Acinetobacter* sp.; BC, *Burkholderia cepacia*; HI, *Haemophilus influenzae*; KP, *Klebsiella pneumoniae*; MC, *Moraxella catarrhalis*; NTM, non‐tuberculosis mycobacterium; PA, *Pseudomonas aeruginosa*; PM, *Pseudomonas pseudomallei*; SA, *Staphylococcus aureus*; SM, *Stenotrophomonas maltophilia*; USG, ultrasound

Patients	Gender	Age at onset of symptom	Age at Dx	Age at time of report	Sweat test results (mmol/L)	Respiratory symptoms	Organism	Latest PFT	MI	PI	Other symptoms	Current status	Mutation 1	Mutation 2
I	Male	2 y.o.	17 y.o.	Succumbed at 23 y.o.	121,126	Chronic productive cough since 2 y.o.; suspected pulmonary TB and treated at 15 y.o.; persistent cough and sputum with a severe superimposed chest infection; HRCT chest at 17 y.o. showed significant bronchiectactic changes at right upper lobe.	**PA**, SA, HI, PM, BC	FEV_1_: 16% pred.	No	No	Soft drug abuser; possibility of lung transplant was rejected as he had BC infection.	Developed progressive respiratory failure, palliative non‐invasive ventilation implemented at 23 y.o. and succumbed 3 months later.	c.1766+5G>T	c.3068T>G: p.I1023R
								FVC: 29% pred.						
								FEV_1_/FVC: 47%						
II	Male	4 m.o.	6 m.o.	14 y.o.	100,112	Two episodes of pneumonia due to SA at 4 and 6 m.o.; chest radiography showed haziness in both lungs; HRCT chest was not done as there has not been any further episodes of significant chest infections.	**SA**, MC, HI	FEV_1_: 78% pred.	No	No	Hyponatraemia, hypokalaemia and metabolic alkalosis at initial presentation; negative screening for adrenal insufficiency; initial growth along 10^th^ percentile.	Height and weight reached 25th percentile; active in various sport activities	c.1766+5G>T	c.3140‐26A>G
								FVC: 79% pred.						
								FEV_1_/FVC: 91%						
III	Male	At birth	2 m.o.	9 y.o.	108,112	Recurrent pneumonia in first 2 years; NTM infection at 5 y.o. and was eradicated; HRCT chest at 5 y.o. showed bronchiectasis, segmental left lingular collapse and patchy parenchymal opacities in both lungs; infrequent respiratory symptoms.	**SA**, Ac, PA, KP, SM, NTM	FEV_1_: 110% pred.	Yes	Yes	Peanut and drug allergy; eczema.	Height and weight at around 10th percentile; pulmonary exacerbations requiring hospitalization about once per year; on antibiotics prophylaxis and airway clearance management.	c.868C>T: p.Q290X	c.3068T>G: p.I1023R
								FVC: 98% pred.						
								FEV_1_/FVC: 83%						
IV	Female	At birth	9 y.o.	12 y.o.	123	Recurrent pneumonia; HRCT chest at 9 y.o. showed significant bronchiectactic changes; persistent cough and sputum.	**PA**, SA	FEV_1_: 68% pred.	Yes	Yes	Abdominal USG showed fatty change in liver; body height at 50th percentile and body weight at 3rd percentile at diagnosis	Height caught up to 90th percentile and weight reached 50th percentile; pulmonary exacerbations requiring hospitalization about once per year; on antibiotics and airway clearance management.	c.1657C>T: p.R533X	c.3068T>G: p.I1023R
								FVC: 74% pred.						
								FEV_1_/FVC: 84%						
V	Female	4 m.o.	13 m.o.	5 y.o.	122,124	Persistent cough since 4 m.o.; recurrent pneumonia and rhinosinusitis; HRCT chest at 13 m.o. showed bronchiectactic changes.	**PA**	Not available as the patient is too young for PFT	No	Yes	Ventricular septal defect which closed spontaneously at 6 m.o.; hyponatraemia, hypokalaemia and hypochloraemia at initial presentation; abdominal USG at 3 y.o. suggested possible liver cirrhosis and early portal hypertension but not confirmed by liver biopsy; liver function remained normal; height was between 10th to 25th percentile and weight was less than 3rd percentile at diagnosis; required supplemental feeding via gastrostomy.	Height caught up to 25th percentile and weight reached 10th percentile since 4 y.o.; oxygen dependent until 4.5 y.o.	c.3068T>G: p.I1023R	c.3068T>G: p.I1023R

Diagnosis was made based on clinical phenotype and abnormal sweat test results after referral to our unit.

a Patients with pancreatic insufficient were given pancreatic enzyme supplement.

### p.I1023R is a *CFTR* founder mutation localized in southern regions of China

**Figure 2:**
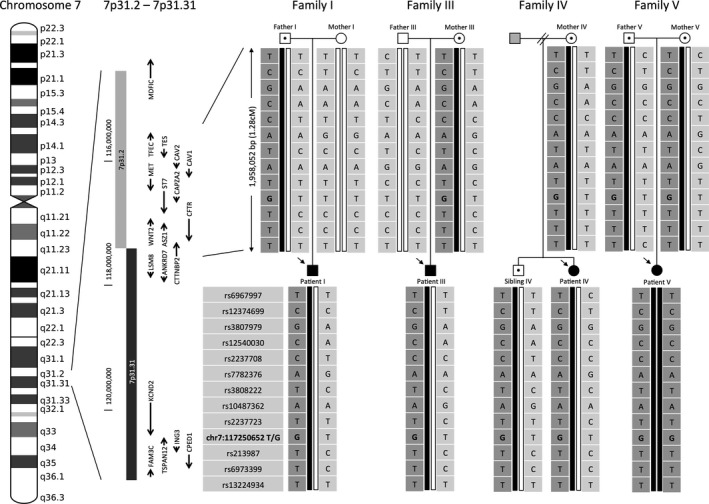
Haplotype analysis of families with the *CFTR* p.I1023R mutation. Pedigrees of the three Hong Kong Chinese (Families I, III and IV) and the Malaysian Chinese (Family V) patients. Arrows mark the index CF patients of each family. The gray symbol represents an individual with an unknown genotype. Open symbols with a dot represent healthy individuals who carry the p.I1023R mutation. p.I1023R is presented as genomic coordinates [hg19]chr7:117250652, which is displayed in bold font. Patient I, patient III, and patient IV are heterozygous for the p.I1023R mutation, whereas index patient V is homozygous for the p.I1023R mutation. The genotypes of selected informative SNPs located closest to the *CFTR* gene are shown directly below the corresponding family members in the pedigrees.

We examined whether the p.I1023R mutation is a founder mutation in the southern Han population. Using genome‐wide single‐nucleotide polymorphism (SNP) microarray genotypic data from four families, we identified a shared haplotype of p.I1023R encompassing 381 SNPs. The estimated physical length of the haplotype is 1,958,052 bp, which is equivalent to a genetic distance of 1.28 cM according to HapMap Phase II (GRCh37/hg19) (Fig. 2). The genomic coordinates of the shared region are chr7:115,513,497–117,471,549. The p.I1023R‐associated haplotype was then compared with the validated control dataset that included 612 local southern Chinese individuals (Yang et al. 2010), who were significantly separated from Chinese Han in Beijing (CHB) HapMap data. We did not find the specific haplotype in any of the control samples. By estimation, the haplotype frequency was less than 4.2 × 10^−5^ (*P*‐value <0.05), and the probability of five independent lineages (from four p.I1023R‐carrier families) by chance sharing the same haplotype was 1.3 × 10^−22^. The p.I1023R allele was exclusively found in the East Asian population at an extremely low frequency of 1.1 × 10^−4^ (1 in 8632 alleles), which suggests its rarity and specificity in East Asians (Exome Aggregation Consortium 2015). We also confirmed the birthplace of p.I1023R‐carrier individuals, and all of the parents were originally from southern provinces of China (Fig. S1). PCA analysis of genome‐wide SNP data (514,006 SNPs) clustered the families with 612 local southern Chinese controls (Fig. S2). These two lines of evidence suggest that all four p.I1023R carrier families are southern Han Chinese. Therefore, the above evidence as well as sibling data from the first report on p.I1023R from Taipei, which is located in the northern region of Taiwan (Ho et al. 2007; Liu et al. 2014), indicate that the mutation is a founder mutation confined to southern regions of China.

### Functional analysis demonstrates that p.I1023R leads to a trafficking defect during CFTR maturation without affecting the gating function of the protein

**Figure 3:**
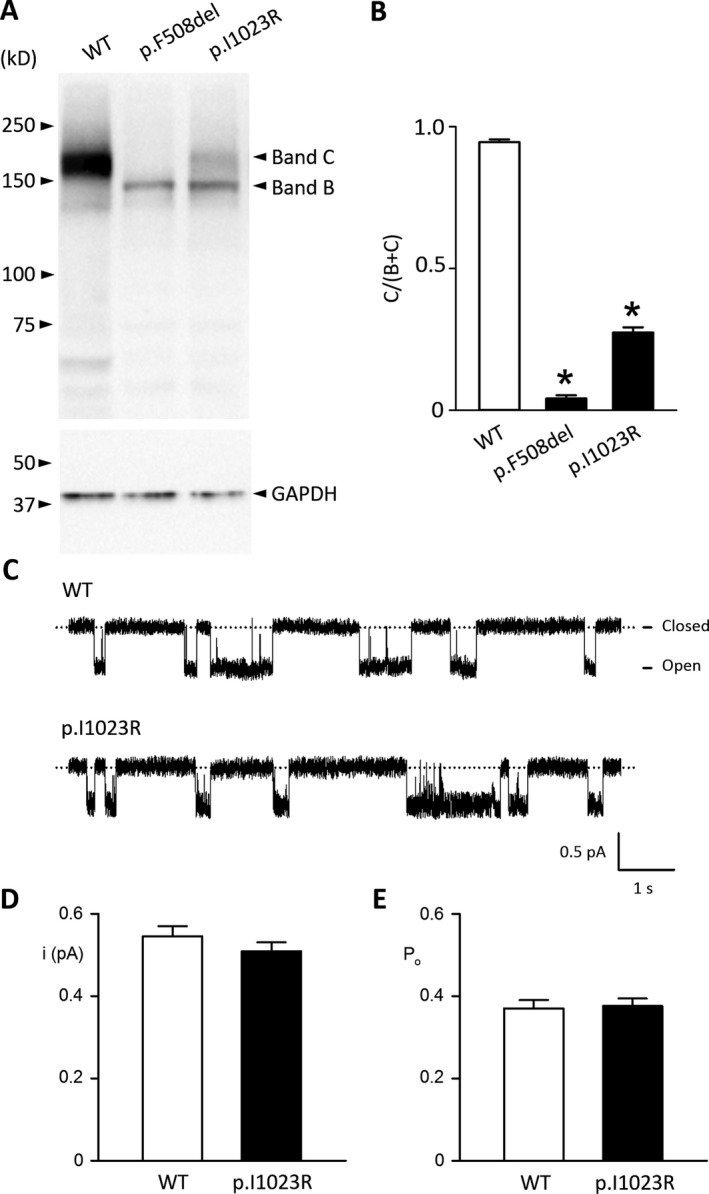
Functional analysis of p.I1023R‐*CFTR*. A. Protein processing of wild‐type CFTR, p.F508del‐CFTR and p.I1023R‐CFTR. The same immunoblotting images are presented. Each lane contains 50 μg total protein. Band C (or C) represents fully glycosylated and mature CFTR, whereas Band B (or B) represents partial glycosylated and immature CFTR. B. The percentage ratio of Band C intensity is normalized to the sum of band B and band C. Data are the mean + SE (*N* = 6). The asterisk indicates that the values between two groups are significantly different (p < 0.001, one‐way anova, Holm‐Sidak method). C. Representative 10 second recordings show the activities of a single wild‐type CFTR and p.I1023R‐CFTR in the excised inside‐out membrane patches at 1 mm ATP and room temperature. The dotted lines indicate closed channels. D and E. The single‐channel current amplitude (*i*) and open probability (*P*_*o*_) are presented. Data are the mean + SE (*N* = 3).

To better understand the molecular defects of the p.I1023R mutant CFTR protein, we investigated protein processing properties by resolving CFTR protein profile, as previously described (Ostedgaard et al. 2007). In this analysis, wild‐type CFTR was characterized by the presence of fully glycosylated proteins was normally transported to the cell membrane, and presented increased expression compared with mutated p.I1023R‐CFTR (Fig. 3A,B), for which a significant proportion of less‐glycosylated protein was found. This finding suggests that the p.I1023R mutation may lead to a trafficking defect, with reduced protein processing from the endoplasmic reticulum to the Golgi apparatus. However, a single‐channel study of p.I1023R‐CFTR using the patch‐clamp method (Chen et al. 2009) demonstrated that the single‐channel current amplitude (*i*) and open probability (*P*_*o*_) of the CFTR chloride channel were not altered by the p.I1023R mutation (Fig. 3C–E). Hence, the dysfunction caused by p.I1023R appears to be largely caused by the reduced expression of fully glycosylated and mature protein.

## Discussion

CF has seldom been reported in Chinese individuals. Here, a molecular diagnosis was performed for four local Chinese patients. Our unit is the only center that offers sweat testing in Hong Kong, a city located in southern China with a population of approximately 7.3 million. We estimated that the incidence of CF is approximately 1 in 300,000 live births in Hong Kong, which is similar to that in Japan (Yamashiro et al. 1997).

We identified the missense mutation p.I1023R in three of four CF patients from Hong Kong as well as in an additional Chinese patient (homozygous) from Malaysia. All four patients with the p.I1023R mutation exhibited had recurrent pneumonia and bronchiectasis. *Pseudomonas aeruginosa* was isolated from all patients, and a more diverse respiratory tract flora was noted in two patients. Three patients had pancreatic insufficiency, and two had meconium ileus. In the limited number of cases presenting with the classical clinical features of CF, we did not observe an association of p.I1023R with a unique clinical phenotype.

The identification of multiple patients with p.I1023R led us to hypothesize that p.I1023R is a potential founder mutation in southern Han Chinese. We defined the following criteria and examined the likelihood of our hypothesis: (1) all patients and p.I1023R carriers share a haplotype associated with the p.I1023R mutation; (2) the p.I1023R‐associated haplotype was shared among affected families with a genetic distance greater than 1 cM; (3) the mutant allele was rare and specific to the population (Fachal et al. 2012); (4) the p.I1023R‐associated haplotype was unique in the normal population; and (5) all carriers were delineated to the same geographic region. Our experimental data were well fit to these criteria well, and we concluded that p.I1023R is indeed a founder mutation in southern Han Chinese. The additional patient from Malaysia who was homozygous for p.I1023R further supported our conclusion. Because the founder haplotype of this mutation was well defined, the time of emergence of p.I1023R can be estimated. Assuming all five p.I1023R lineages from four families to be independent, all of the patients from this study inherited the mutation from a common ancestor approximately 15.6 generations (i.e., meiotic events) ago.

Although p.I1023R is the most common *CFTR* mutation in our cohort, it was not observed in the cohort of 20 CF patients recently reported by investigators from Beijing (Liu et al. 2015; Tian et al. 2016). Another Chinese‐specific mutation, p.G970D, appears to be more commonly observed in northern Chinese with CF. We summarize our findings along with previously reported *CFTR* mutations. Interestingly, an obvious difference is observed in the spectrum of *CFTR* mutations between the northern and southern regions of China (Fig. 1). Although further studies must be performed to elucidate the complete picture of *CFTR* molecular epidemiology in China, current evidence suggests that compared with Caucasians, Chinese CF patients have a different *CFTR* mutation spectrum. As translating CF molecular tests into clinical practice, such as through the use of a 23‐variant ACMG CF panel (Watson et al. 2004), will result in a low diagnostic yield when applied to Chinese patients, a customized Chinese‐specific CF panel may be required to investigate the incidence of CF in Chinese patients. This Chinese‐specific CF panel is not only relevant for Asian countries but also for other parts of the world that present significant population of Chinese immigrants. For example, the 2011 US Census reported that the Chinese American population included approximately 3.8 million individuals and accounted for 1.2% for the overall population.

Here, we illustrate that p.I1023R causes a disruption in CFTR intracellular processing, though the conductance (*i*) and channel open probability (*P*_*o*_) remained similar to that of the wild‐type protein. Our calculation of normal CFTR expression (i.e., 25%) is likely an overestimate (Moran and Zegarra‐Moran 2008). We tested expression of the mutant protein via the overexpression of foreign plasmids in a cell line and analyzing the total protein content from the cell extract, including the CFTR protein trapped in the Golgi apparatus and cell membrane. However, the band intensity ratio did not reflect the actual proportion of functional CFTR in the cell membrane compared with wild‐type CFTR. In addition, we did not define an association between the physiological values of p.I1023R‐associated CFTR expression and a clinical phenotype of CF. Moreover, our data clearly indicate that muturation of the p.I1023R‐CFTR protein maturation was inhibited.

In general, genetic mutations primarily disrupt CFTR functions by reducing protein expression or channel activity. We functionally characterized p.I1023R as a class II *CFTR* mutation (reducing protein expression) based on the functional properties of the gene product as originally proposed by Tsui (1992). Similar to p.I1023R, several other mutations, such as p.A561E, p.S1235R, and p.N1303K, are also to class II mutations according to their pathological mechanism (Welsh and Smith 1993; Mendes et al. 2003). Understanding the pathological mechanisms of each class of mutation can shed light on the development of mutation‐specific therapies. For instance, the protein maturation process affected by p.A561E was enhanced by lumacaftor treatment (Awatade et al. 2014), whereas human cell lines with p.S1235R and p.N1303K responded positively to treatment with small molecular correctors (Rapino et al. 2015). Thus, our findings can be used as the basis for future clinical trials in drug development.

To conclude, we provide the first report of a founder mutation, p.I1023R, in *CFTR* in southern Chinese individuals as well as a functional characterization of this mutation. Because this mutation and other known mutations found in Chinese patients are not currently included in the conventional *CFTR* gene panel for the molecular diagnosis of CF, we suggest a different *CFTR* gene panel or mutation screening strategy for CF patients of Chinese origin.

## Conflict of Interest

The authors declare that they have no actual or potential conflicts of interest, including any financial, personal or other relationships with people or organizations regarding the submitted work.

## Supporting information


**Figure S1.** Birthplace of the individuals with p.I1023R mutation involved.
**Figure S2.** Principal components analysis (PC1 vs. PC2) of 612 Hans Chinese (gray dots) from Hong Kong and 12 individuals from the affected family with p.I1023R (black dots).
**Figure S3.** Target coverage of the childhood bronchiectasis panel. The panel includes 16 selected candidate genes for primary ciliary dyskinesia (PCD) and cystic fibrosis (CF).
**Table S1.** A list of reported CF Chinese patients with *CFTR* mutations identified.
**Appendix S1.** Experimental workflow.
