# Supplementary material for: CFTR founder mutation causes protein trafficking defects in Chinese patients with cystic fibrosis
Source: Mol Genet Genomic Med. 2016 Nov 13;5(1):40–9. doi: 10.1002/mgg3.258 (PMC5241212; doi:10.1002/mgg3.258)
Supplement: Supplementary file 1 — Figure S1. Birthplace of the individuals with p.I1023R mutation involved. Figure S2. Principal components analysis (PC1 vs. PC2) of 612 Hans Chinese (gray dots) from Hong Kong and 12 individuals from the affected family with p.I1023R (black dots). Figure S3. Target coverage of the childhood bronchiectasis panel. The panel includes 16 selected candidate genes for primary ciliary dyskinesia (PCD) and cystic fibrosis (CF). Table S1. A list of reported CF Chinese patients with CFTR mutations identified. Appendix S1. Experimental workflow. [file MGG3-5-40-s001.docx]

Authors: Leung GKC^1^, Ying D^2^, Mak CCY^1^, Chen XY^3^, Xu W^3^, Yeung KS^1^, Wong WL^1^, Chu YWY^1^, Mok GTK^1^, Chau CSK^4^, McLuskey J^5^, Ong WPT^6^, Leong HY^6^, Chan KYK^7^, Yang W^1^, Chen JH^3^, Li AM^8^, Sham PC^2^, Lau YL^1^, Lee SL^1^*, Chung BHY^1^*

Title: CFTR founder mutation causes protein trafficking defects in Chinese patients with cystic fibrosis

Affiliations:

^1^ Department of Paediatrics and Adolescent Medicine, LKS Faculty of Medicine, The University of Hong Kong, HKSAR; ^2^ Department of Psychiatry, LKS Faculty of Medicine, The University of Hong Kong, HKSAR; ^3^ HKU Shenzhen Institute of Research and Innovation, School of Biomedical Sciences, The University of Hong Kong, HKSAR; ^4^ Department of Paediatrics and Adolescent Medicine, Queen Mary Hospital, HKSAR; ^5^ NHS Lothian, Edinburgh, Scotland, UK; ^6^ Department of Genetics, Kuala Lumpur Hospital, Kuala Lumpur, Malaysia; ^7^ Department of Obstetrics and Gynaecology, Tsan Yuk Hospital, HKSAR; ^8^ Department of Paediatrics, Faculty of Medicine, The Chinese University of Hong Kong, HKSAR

e-Fig. 1: Birthplace of the individuals with p.I1023R mutation involved. The cross indicates the geographic location of Hong Kong. Each silhouette represents one p.I1023R carrier. The siblings from the first p.I1023R report in Taiwan were also included. Silhouettes with a number refer to the corresponding CF patients in this study. The black circles with a number refer to the parent with p.I1023R mutation.


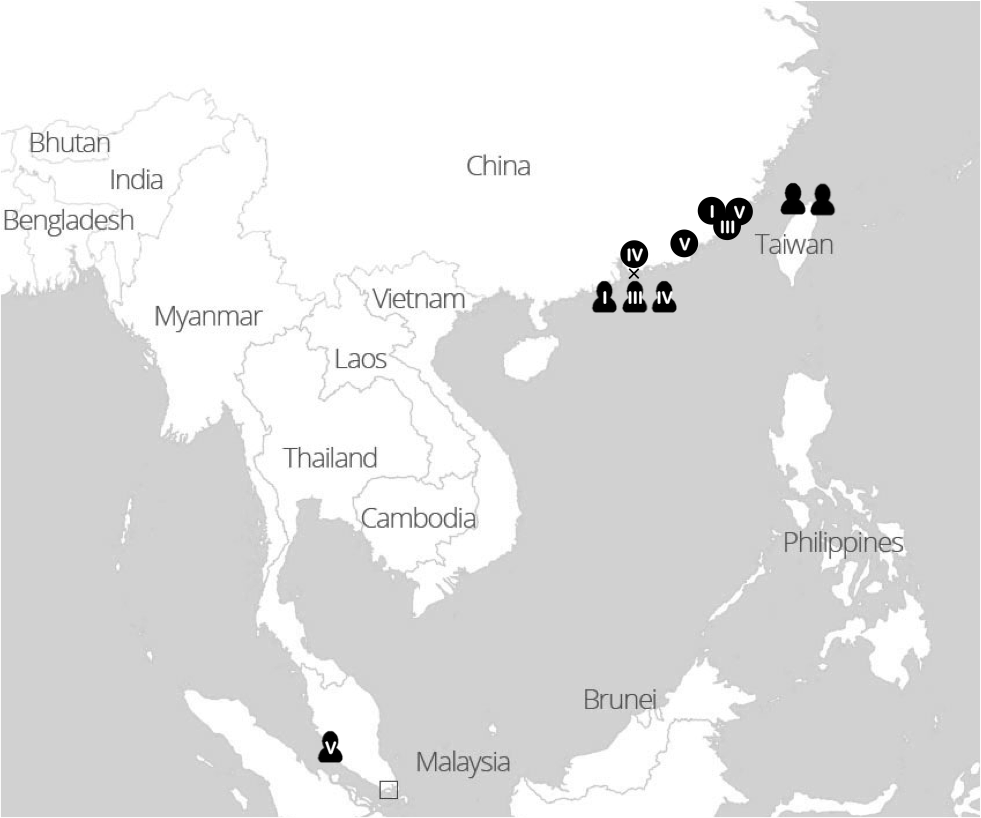


e-Fig. 2: Principal components analysis (PC1 versus PC2) of 612 Hans Chinese (grey dots) from Hong Kong and 12 individuals from the affected family with p.I1023R (black dots). The x-axis represents principal component 1 (PC1) and the y-axis represents principal component 2 (PC2).
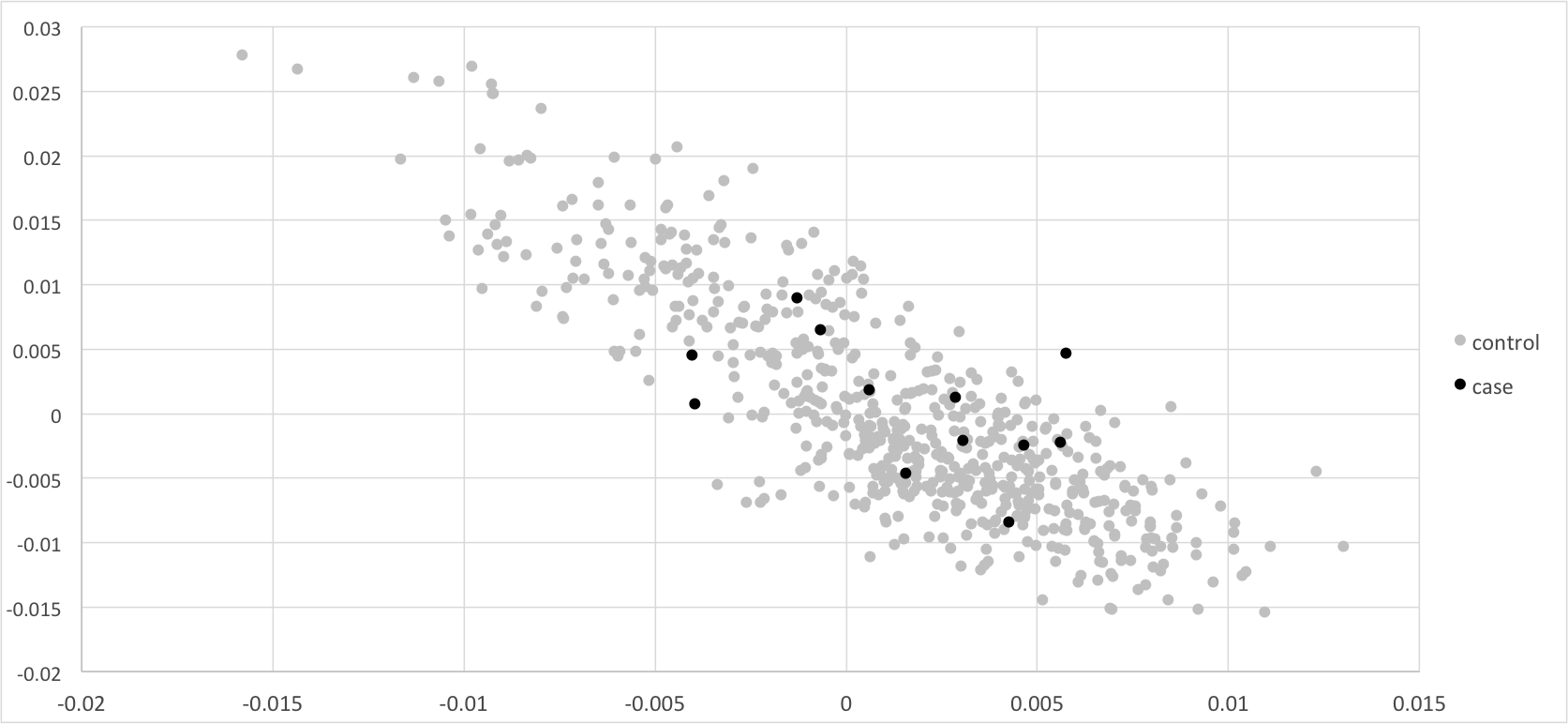
The graph was generated from all 514,006 SNPs shared among all the individuals. All members from the affected families were clustered within the local Chinese population, which has been validated as Southern Chinese, suggesting that the individuals involved in the linkage analysis were all of Hans Chinese ethnicity.

e-Fig. 3: Target coverage of the childhood bronchiectasis panel. The panel includes 16 selected candidate genes for primary ciliary dyskinesia (PCD) and cystic fibrosis (CF).


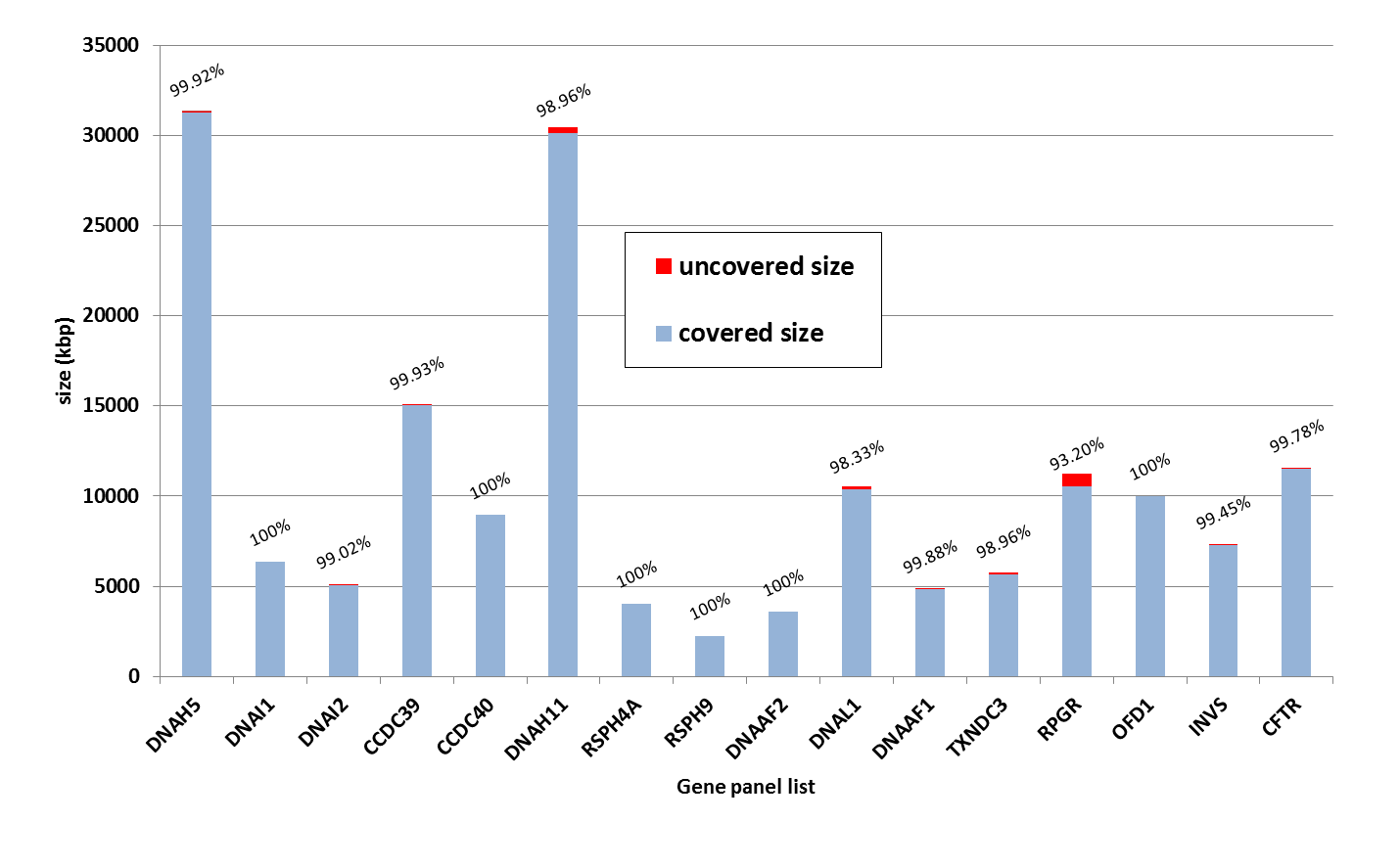

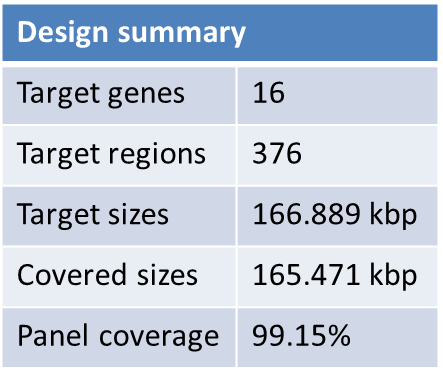


e-Table. 1: A list of reported CF Chinese patients with *CFTR* mutations identified. The first five patients were described in this manuscript. Only patients with both diseasing-causing mutations identified were listed. HGVS nomenclature is based on the naming practice developed by the Human Genome Variation Society. Mutations with an asterisk indicate the genetic variant was first reported in the corresponding literatures; n.a. = data not available; Chi/Vie = patient from both Chinese and Vietnam lineage.

|  |  |  |  |  | **Sweat Chloride (mmol/L)** | |  | **Allele 1** | |  | **Allele 2** | |  |
| --- | --- | --- | --- | --- | --- | --- | --- | --- | --- | --- | --- | --- | --- |
| **Patient** | **Gender** | **Con-**  **sanguinity** | **Ethnicity** | **Age**  **at Dx** | **1st** | **2nd** |  | **Reported name** | **HGVS nomenclature** |  | **Reported name** | **HGVS nomenclature** | **Reference** |
| 1 | M | No | Chinese | 17 years | 121 | 126 |  | c.1766+5G>T | c.1766+5G>T |  | c.3068T>G | c.3068T>G:p.I1023R | This study |
| 2 | M | No | Chinese | 6 months | 100 | 112 |  | c.1766+5G>T | c.1766+5G>T |  | c.3140-26A>G | c.3140-26A>G | This study |
| 3 | M | No | Chinese | 2 months | 108 | 112 |  | c.868C>T | c.868C>T:p.Q290X |  | c.3068T>G | c.3068T>G:p.I1023R | This study |
| 4 | F | No | Chinese | 9 years | 123 | n.a. |  | c.1657C>T | c.1657C>T:p.R533X |  | c.3068T>G | c.3068T>G:p.I1023R | This study |
| 5 | F | No | Chinese | 13 months | 122 | 124 |  | c.3068T>G | c.3068T>G:p.I1023R |  | c.3068T>G | c.3068T>G:p.I1023R | This study |
| 6 | M | n.a. | Chinese | 20 years | 137 | n.a. |  | c.2909G>A | c.2909G>A:p.G970D |  | c.1521_1523delCTT | c.1521_1523delCTT:p.F508del | (Tian et al., 2016) |
| 7 | F | n.a. | Chinese | 15 years | 140 | n.a. |  | c.2909G>A | c.2909G>A:p.G970D |  | c.2374C>T | c.2374C>T:p.R792X | (Tian et al., 2016) |
| 8 | F | n.a. | Chinese | 1 year | 108.4 | n.a. |  | c.2909G>A | c.2909G>A:p.G970D |  | c.2125C>T | c.2125C>T:p.R709X | (Tian et al., 2016) |
| 9 | M | n.a. | Chinese | 13 years | 95.2 | n.a. |  | c.3700A>G | c.3700A>G:p.I1234V |  | c.959-960insA | c.959-960insA:p.S321IfsX42 | (Tian et al., 2016) |
| 10 | F | n.a. | Chinese | 22 years | 101.9 | n.a. |  | c.2909G>A | c.2909G>A:p.G970D |  | c.1997T>G | c.1997T>G:p.L666X | (Tian et al., 2016) |
| 11 | F | n.a. | Chinese | 4 years | 122.1 | n.a. |  | c.2909G>A | c.2909G>A:p.G970D |  | c.263T>G | c.263T>G:p.L88X | (Tian et al., 2016) |
| 12 | F | n.a. | Chinese | 13 years | 62 | n.a. |  | c.2909G>A | c.2909G>A:p.G970D |  | c.2907A>C | c.2907A>C:p.A969A | (Tian et al., 2016) |
| 13 | F | Yes | Chinese | 11 years | 154 | n.a. |  | c.293A>G | c.293A>G:p.Q98R |  | c.293A>G | c.293A>G:p.Q98R | (Liu et al., 2015) |
| 14 | M | No | Chinese | New-born | 66 | n.a. |  | c.95T>C | c.95T>C:p.L32P |  | c.1657C>T | c.1657C>T:p.R533X | (Liu et al., 2015) |
| 15 | M | No | Chinese | 1 year | 135 | n.a. |  | c.293A>G | c.293A>G:p.Q98R |  | c.558C>G | c.558C>G:p.N186K | (Liu et al., 2015) |
| 16 | M | No | Chinese | New-born | 132 | n.a. |  | c.2052_dupA | c.2052_dupA:p.Q686TfsX3 |  | △E18-E20 | c.2909?_3367+?del:p.G980_T1112delinsG | (Liu et al., 2015) |
| 17 | F | No | Chinese | 11 years | 130 | n.a. |  | c.2909G>A | c.2909G>A:p.G970D |  | △E7-E11 | c.744?_1584+?del:p.R248_E528delinsRfsX11 | (Liu et al., 2015) |
| 18 | F | No | Chinese | 6 years | 156 | n.a. |  | c.1679+2T>C | c.1679+2T>C |  | c.2658-1G>C | c.2658-1G>C | (Liu et al., 2015) |
| 19 | F | No | Chinese | 20 years | n.a. | n.a. |  | 3849+10kb C>T | c.3717+12191C>T |  | 3849+10kb C>T | c.3717+12191C>T | (Liu et al., 2014) |
| 20 | F | n.a. | Chinese | 13 years | elevated | n.a. |  | 263T>G | c.263T>G:p.L88X |  | 2909G>A | c.2909G>A:p.G970D | (Liu and Kang, 2012) |
| 21 | F | No | Chinese | 10 years | n.a. | n.a. |  | 3196 C>T | c.3196C>T:p.R1066C |  | 3196C>T | c.3196C>T:p.R1066C | (Liu and Kang, 2012) |
| 22 | F | No | Chinese | 12 years | 123.6 | 108 |  | W679X | c.2036G>A:p.W679X |  | 3120+2T>C | c.2988+2T>C | (Cheng et al., 2012) |
| 23 | M | No | Chinese | 16 years | 100 | n.a. |  | 1898+5G>T | c.1766+5G>T |  | *I1023R | c.3068T>G:p.I1023R | (Ho et al., 2007) |
| 24 | M | No | Chinese | 14 years | elevated | 65 |  | 1898+5G>T | c.1766+5G>T |  | I1023R | c.3068T>G:p.I1023R | (Ho et al., 2007) |
| 25 | F | No | Chinese | 14 years | 108.9±3.3 (mean+ SD) | |  | 699C>A | c.567C>A:p.N189K |  | 3821-3823 delT | c.3691delT : p.S1231PfsX4 | (Li et al., 2006) |
| 26 | M | No | Chinese | < 1 year | n.a. | n.a. |  | R533X | c.1657C>T:p.R533X |  | R533X | c.1657C>T:p.R533X | (Chen et al., 2005) |
| 27 | M | No | Chi/Vie | 1.5 years | 89 | n.a. |  | G151T | c.19G>T:p.E7X |  | 989-992insA | c.857_858insA:p.N287LfsX21 | (Wong et al., 2003) |
| 28 | F | No | Chinese | 6 months | 135 | n.a. |  | 1898+5G>T | c.1766+5G>T |  | 2215insG and S895N | c.[2083_2084insG; 2684G>A];p.[E695GfsX35; S895N] | (Wang et al., 1993, Alper et al., 2003) |
| 29 | M | No | Chinese | 17 years | 327 | n.a. |  | 1898+5G>T | c.1766+5G>T |  | 2215insG and S895N | c.[2083_2084insG; 2684G>A];p.[E695GfsX35; S895N] | (Wu et al., 2000) |
| 30 | F | No | Chinese | 14 years | 276 | n.a. |  | 1898+5G>T | c.1766+5G>T |  | 2215insG and S895N | c.[2083_2084insG; 2684G>A];p.[E695GfsX35; S895N] | (Wu et al., 2000) |
| 31 | F | No | Chinese | 23 years | 104 | n.a. |  | 451-458 8bp del | c.319_326delGCTTCCTA:p.A107X |  | G3041A | c.2909G>A:p.G970D | (Wagner et al., 1999) |
| 32 | F | Yes | Chinese | 8 years | elevated | n.a. |  | 1898+5G>T | c.1766+5G>T |  | 1898+5G>T | c.1766+5G>T | (Zielenski et al., 1995) |

e-appendix:

Experimental workflow:

*An Integrated approach for molecular diagnosis*

Genomic DNA was extracted from peripheral blood using standard protocol. A customized gene enrichment panel for childhood onset bronchiectasis was designed and used in our next-generation sequencing-based diagnostic procedure. The panel covers the exons (flanked by ± 25 bp) of 16 selected candidate genes for primary ciliary dyskinesia (PCD) and CF (e-Fig.4). Only findings related to *CFTR* are described here. Target enrichment was done using Haloplex custom target enrichment panel according to the manufacturer protocol with a DNA input of 2.5 μg (Agilent Technologies, CA). Sequencing was done on the MiSeq platform with MiSeq reagent kit V2 (Illumina Inc., CA). Raw reads were filtered, aligned and called using NextGENe software v.2.3.4.1 (Softgenetics, PA). Sanger sequencing was used for (i) re-sequencing of low coverage regions, defined as region of read depth <20X, and (ii) validation of pathogenic variants from patients and parents.

*Haplotype analysis using genome-wide SNP microarray*

In addition to the patients recruited in Hong Kong, a Chinese CF patient from Malaysia with homozygous p.I1023R and her carrier parents were referred to us for haplotype analysis. We genotyped the first-degree family members of CF patients with p.I1023R, and determined whether they are the carrier of the mutation using Sanger sequencing. In total, twelve individuals from 4 independent affected families were involved in our haplotype analysis. This includes 10 individuals with p.I1023R (4 probands, 5 parents and 1 carrier sibling), as well as 2 non-p.I1023R carriers from different families. We genotyped the target individuals by using HumanOmniZhongHua-8 BeadChip (Illumina Inc., CA). We delineated a preliminary shared region of 2.4 Mb based on the preliminary SNP and STR markers analysis (data not shown). Data was then processed using GenomeStudio (Illumina Inc., CA). Principal component analysis was performed using EIGENSTRAT software package. A total of 479 SNPs were analysed.

Genotype data of 612 healthy Southern Han Chinese control individuals were involved in our analysis (Yang et al., 2010). All of them were of self-reported Chinese living in Hong Kong. They were genotyped using the llumina 610-Quad Human Beadchip (Illumina Inc., CA). Within the defined haplotype of our interest, 130 SNPs from the control data were shared with the SNPs data of the p.I1203R carrier families. The data was used in principal component analysis for population segregation and haplotype frequency segregation.

*Characterization of glycosylation level in p.I1023R protein using protein electrophoresis*

The wild-type-CFTR/pcDNA3.1 and the mutant p.I1023R-CFTR/pcDNA3.1 expression plasmids were generated by site-directed mutagenesis kit (Agilent Technologies, CA) and verified by Sanger sequencing. HeLa cells were transfected with the wild-type and mutant plasmids using lipofectamine 2000 (Invitrogen, MA).

Transfected cells were washed with ice-cold PBS and then lysed in buffer with cOmplete™ protease inhibitors (Roche, Switzerland). Supernatant from the lysate were then quantified using Quick start Bradford protein assay (BioRad, CA). Protein (50 μg) separation was conducted using 6% SDS-PAGE gel electrophoresis at 150 V for 4 hours. Separated proteins were transferred to a PVDF membrane at a voltage of 30 V in 4^o^C overnight. The PVDF membrane was sequentially blocked with 0.1% Casein (Sigma-Aldrich, MO), incubated with 1:1,000 CFTR antibodies M3A7, MM13-4 and MM13-1 (Merck Millipore, MA). The membrane was washed with TBST before incubation with 1:3,000 goat anti-mouse HRP antibody (Merck Millipore, MA) and washed again with TBST before chemiluminescence detection using WesternBright ECL reagents (Advasta, CA). Images were captured using ChemiDoc XRS system (BioRad, CA) and analyzed by the software Image J. Data is presented as means + SEM. Differences in band intensity between two groups were considered statistically significant at *P* < 0.05, one-way ANOWA.

*Determination of gating activity using patch-clamp test*

HeLa cells transfected with wild-type and mutant plasmids were used. Single-channel currents in the excised inside-out membrane patches were recorded. The CFTR activity in the membrane patches was activated and maintained by adding ATP (1 mM) and PKA (75 nM) in the intracellular solution at room temperature. Membrane potential was clamped at -50 mV to amplify current amplitude. Single-channel currents were recorded by an Axopatch 200B amplifier (Molecular Devices, CA), filtered at 500 Hz with a 900C eight-pole bessel filter (Frequency Devices, IL) and digitized at 10 KHz by a Digidata 1440A digitizer (Molecular Devices, CA). To measure the single-channel activity of the CFTR channel, the single-channel current amplitude (*i*) was obtained from the fit of Gaussian distribution to the single-channel current amplitude histograms. The open probability (*P_o_*) was derived by the event lists of current recordings from the membrane patches containing four or less activated channels. Current recording and data analysis were performed by pClamp 10 software.

Reference list

ALPER, O. M., SHU, S. G., LEE, M. H., WANG, B. T., LO, S. Y., LIN, K. L., CHIU, Y. L. & WONG, L. J. 2003. Detection of novel CFTR mutations in Taiwanese cystic fibrosis patients. *Journal of the Formosan Medical Association,* 102**,** 287-91.

CHEN, H. J., LIN, S. P., LEE, H. C., CHEN, C. P., CHIU, N. C., HUNG, H. Y., CHERN, S. R. & CHUANG, C. K. 2005. Cystic fibrosis with homozygous R553X mutation in a Taiwanese child. *J Hum Genet,* 50**,** 674-8.

CHENG, Y., NING, G., SONG, B., GUO, Y. & LI, X. 2012. A Chinese girl with cystic fibrosis: a case report identified by sweat and genetic tests. *Chinese medical journal,* 125**,** 719-719.

HO, Y., WU, W., WONG, K., HUANG, C., NIU, C., SHYUR, S., HUANG, L. & WU, P. 2007. Novel Mutation (I1023R) in Two Taiwanese Siblings of Cystic Fibrosis. *中华民国儿童胸腔医学会杂志 [Journal of Pediatric Respiratory Disease],* 5**,** 152-158.

LI, N., PEI, P., BU, D., HE, B. & WANG, G. 2006. A novel CFTR mutation found in a Chinese patient with cystic fibrosis. *Chinese medical journal,* 119**,** 103-109.

LIU, L., SHYUR, S., CHU, S., HUANG, L., KAO, LEI, W., CHENG, C., LO, C., CHEN, C. & FANG, L. 2014. Cystic fibrosis: Experience in one institution. *Journal of Microbiology, Immunology and Infection,* 47**,** 358-361.

LIU, T. & KANG, J. 2012. [Analysis of Clinical Features of Cystic Fibrosis in Chinese]. *[Journal of Chinese General Practice],* 15**,** 2807-10.

LIU, Y., WANG, L., TIAN, X., XU, K. F., XU, W., LI, X., YUE, C., ZHANG, P., XIAO, Y. & ZHANG, X. 2015. Characterization of gene mutations and phenotypes of cystic fibrosis in Chinese patients. *Respirology,* 20**,** 312-8.

TIAN, X., LIU, Y., YANG, J., WANG, H., LIU, T., XU, W., LI, X., ZHU, Y., XU, K.-F. & ZHANG, X. 2016. p. G970D is the most frequent CFTR mutation in Chinese patients with cystic fibrosis. *Human Genome Variation,* 3.

WAGNER, J. A., VASSILAKIS, A., YEE, K., LI, M., HURLOCK, G., KROUSE, M. E., MOSS, R. B. & WINE, J. J. 1999. Two novel mutations in a cystic fibrosis patient of Chinese origin. *Hum Genet,* 104**,** 511-5.

WANG, M. C., SHU, S. G., CHANG, S. M., HO, W. L. & CHI, C. S. 1993. Cystic fibrosis in two Chinese infants in Taiwan. *Zhonghua Min Guo Xiao Er Ke Yi Xue Hui Za Zhi,* 34**,** 314-21.

WONG, L. J., ALPER, O. M., WANG, B. T., LEE, M. H. & LO, S. Y. 2003. Two novel null mutations in a Taiwanese cystic fibrosis patient and a survey of East Asian CFTR mutations. *Am J Med Genet A,* 120a**,** 296-8.

WU, C. L., SHU, S. G., ZIELENSKI, J., CHIANG, C. D. & TSUI, L. C. 2000. Novel cystic fibrosis mutation (2215insG) in two adolescent Taiwanese siblings. *J Formos Med Assoc,* 99**,** 564-7.

YANG, W., SHEN, N., YE, D. Q., LIU, Q., ZHANG, Y., QIAN, X. X., HIRANKARN, N., YING, D., PAN, H. F., MOK, C. C., CHAN, T. M., WONG, R. W., LEE, K. W., MOK, M. Y., WONG, S. N., LEUNG, A. M., LI, X. P., AVIHINGSANON, Y., WONG, C. M., LEE, T. L., HO, M. H., LEE, P. P., CHANG, Y. K., LI, P. H., LI, R. J., ZHANG, L., WONG, W. H., NG, I. O., LAU, C. S., SHAM, P. C., LAU, Y. L. & ASIAN LUPUS GENETICS, C. 2010. Genome-wide association study in Asian populations identifies variants in ETS1 and WDFY4 associated with systemic lupus erythematosus. *PLoS Genet,* 6**,** e1000841.

ZIELENSKI, J., MARKIEWICZ, D., LIN, S. P., HUANG, F., YANGFENG, T. L. & TSUI, L. C. 1995. Skipping of exon 12 as a consequence of a point mutation (1898 + 5G→T) in the cystic fibrosis transmembrane conductance regulator gene found in a consanguineous Chinese family. *Clinical genetics,* 47**,** 125-132.
